# Dengue virus infection increases microglial cell migration

**DOI:** 10.1038/s41598-017-00182-z

**Published:** 2017-03-07

**Authors:** Ming-Kai Jhan, Tsung-Ting Tsai, Chia-Ling Chen, Cheng-Chieh Tsai, Yi-Lin Cheng, Yi-Chao Lee, Chiung-Yuan Ko, Yee-Shin Lin, Chih-Peng Chang, Liang-Tzung Lin, Chiou-Feng Lin

**Affiliations:** 1 Graduate Institute of Medical Sciences, College of Medicine, Taipei Medical University, Taipei, 110 Taiwan; 2 Department of Microbiology and Immunology, School of Medicine, College of Medicine, Taipei Medical University, Taipei, 110 Taiwan; 3 Translational Research Center, Taipei Medical University, Taipei, 110 Taiwan; 4 Department of Nursing, Chung Hwa University of Medical Technology, Tainan, 717 Taiwan; 5 Institute of Basic Medical Science, College of Medicine, National Cheng Kung University, Tainan, 701 Taiwan; 6 The Ph.D. Program for Neural Regenerative Medicine, College of Medical Science and Technology, Taipei Medical University, Taipei, 110 Taiwan; 7 Department of Microbiology and Immunology, College of Medicine, National Cheng Kung University, Tainan, 701 Taiwan; 8 Center of Infectious Diseases and Signaling Research, National Cheng Kung University, Tainan, 701 Taiwan

## Abstract

Activated microglial cells are present in dengue virus (DENV)-infected brains; however, the possible effects of DENV on microglia remain unclear. Here, we demonstrated DENV caused infection, including viral entry, RNA replication, viral protein expression, and virus release, in the murine microglial cell line BV2. DENV infection caused an increase in the formation of the multipolar phenotype *in vitro* and *in vivo* without affecting cell growth and cytotoxicity. DENV infection considerably increased cell motility and disrupting either actin filaments or clathrin retarded such effect. Increase in cell migration was only occurred by DENV infection following a clathrin-regulated endocytosis of DENV entry. Ultraviolet-inactivated DENV did not affect cell migration, and pharmacologically blocking toll-like receptor (TLR) 3 and TLR3-related signaling pathways reduced the DENV-induced increase in cell migration. These results demonstrate an advanced effect of DENV infection on microglial migration via a mechanism involving viral entry, RNA release, and TLR3 signal activation.

## Introduction

Dengue virus (DENV) infection causes mild dengue fever, which is an arthropod-borne viral disease that accounts for approximately 95% of all reported dengue cases, as well as severe dengue^1^. The definition of severe dengue in patients has been guided by the presence of many features, including plasma leakage, bleeding, consciousness, severe gastrointestinal and organ impairment, and other unusual manifestations^1^. The case fatality rate in severe dengue ranges between 1 and 10% depending on early recognition and proper treatment. A study of fatal patients showed higher frequencies of early altered consciousness (≤24 h after hospitalization), hypothermia, bleeding, shock, concurrent bacteremia, pulmonary edema, renal/hepatic failure, and subarachnoid hemorrhage^2^. During CNS infection, severe dengue patients may exhibit neurological complications, including dengue encephalopathy, encephalitis, neuromuscular complications, and neuro-ophthalmic involvement^3^. Although the viral genome, proteins, and particles can be detected in the brains of fatal dengue patients^4–6^ and experimentally infected mice^7, 8^, the targeting of DENV-infected cells and their effects on neurotoxicity and brain dysfunction have not been well explored.

DENV was reported to infect cells in the brain following blood-brain barrier (BBB) destruction in a murine model of DENV infection-induced encephalitis following concurrent intracerebral and intraperitoneal inoculation^9^. Another study using intraperitoneal inoculation of DENV infection showed BBB damage followed by plasma leakage in the brain^8^. However, this model utilized an adapted neuroinvasive and neurovirulent strain of DENV. Interestingly, a current study reported antibody-dependent enhancement of DENV infection in the brain in a *Callithrix penicillata* monkey, followed by the induction of severe CNS inflammation characterized by cytokine overproduction and microglial cell activation^10^. However, whether DENV infection directly or indirectly damages the BBB is unclear. Activated microglia, which are resident macrophage-like immune cells in the brain, are widely present in neurological disorders including infection and may act as amplifiers for neuroinflammation^11^. Regarding the role of monocytes/macrophages as targets of DENV infection^12–15^, an *in vitro* study demonstrated that DENV infected and activated the microglial cell line BV2 by inducing the transcriptional activation of several inflammatory cytokines^16^. Based on the *in vitro* and *in vivo* results, microglia can be the targets of DENV in the brain; however, the effects of DENV on microglia require further investigation.

Following the binding of cellular receptors to the DENV envelope protein, there are distinct entry pathways for DENV internalization, including clathrin-mediated and clathrin-independent endocytosis, depending on the host cell and virus serotype^15, 17^. Upon clathrin-mediated entry, DENV particles are actively transported into the endosomes and then fuse with the endosomal membrane to release viral RNA under endosomal acidification^18^. Although viral RNA redistributes to the endoplasmic reticulum, the DENV ssRNA is immediately translated into viral proteins (especially non-structural proteins) to facilitate dsRNA replication followed by assembly of the viral particles with structural proteins^19^. To date, no reports have shown the entry pathway and the effects of DENV on microglia. In this report, we demonstrated that DENV caused infection, including viral binding, entry, dsRNA replication, viral protein expression, and virus release, in microglial BV2 cells. Following DENV infection, clathrin-mediated endocytosis signaling followed by TLR3 activation induced an increase in microglial migration. We also investigated the molecular mechanisms involved in these processes and demonstrated the involvement of TLR3-related signaling pathways.

## Results

### DENV initiates infection in microglia *in vitro*, including viral entry, RNA replication, protein expression, and viral release

**Figure 1:**
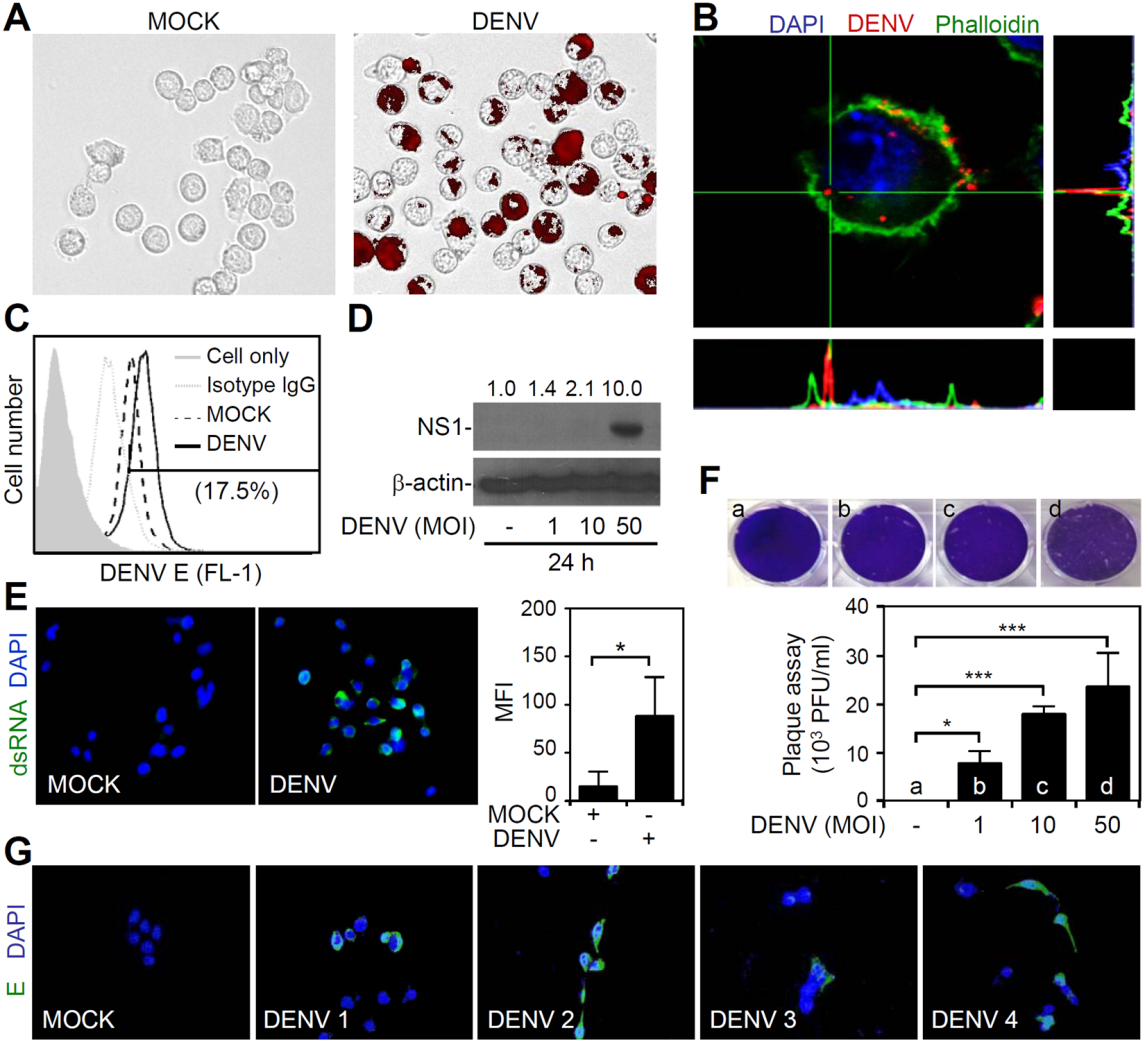
DENV causes infection, including entry, viral protein expression, and replication, in the microglial cell line BV2. Microglial BV2 cells were inoculated with Alexa-594 labeled (*red*) DENV serotype2 PL046 (MOI = 50) for 2 h. (**A**) Fluorescent image analysis showed microglial cells carrying fluorescent DENV 2. (**B**) Three-dimensional confocal images confirmed viral entry. Phalloidin (*green*) staining indicated actin. (**C**) Flow cytometry analysis demonstrated viral envelope (*E*) protein expression 24 h post-infection. (**D**) Western blotting analysis showed viral nonstructural protein1 (*NS1*) expression in DENV-infected cells. The relative ratio to β-actin is shown. (**E**) Immunocytochemistry and its relative mean fluorescence intensity (*MFI*) of viral dsRNA 24 h post-infection. (**F**) Plaque assays showed the level of viral replication. (**G**) Immunocytochemistry showed viral E protein expression (*green*) in cells infected with the four DENV serotypes. For all images and blots, representative data were selectively obtained from three individual experiments. DAPI staining indicated the nuclei (*blue*). For the flow cytometry analysis, the percentage of positive cells is shown. All quantitative data are shown as the means ± SD from three independent experiments. *p < 0.05, **p < 0.01, and ***p < 0.001.

Our current study^20^ and the previous works^8^ have showed that DENV can infect CD11b-positive or Iba-1-positive microglial cells *in vivo*; however, the effects of DENV on microglia remain poorly understood. This study investigated the effects of DENV serotype 2 PL046 on the murine microglial cell line BV2. To demonstrate the infectious efficacy, we performed fluorescent DENV staining followed by fluorescent imaging (Fig. 1A) and flow cytometric analysis (data not shown). The results showed viral binding/entry in BV2 cells 2 h post-inoculation. To confirm this finding, we performed confocal microscopy to evaluate the intracellular localization of fluorescence-stained DENV in BV2 cells (Fig. 1B). To investigate the replication of DENV in microglial cells, we performed flow cytometry (Fig. 1C) and Western blotting (Fig. 1D). Our results confirmed viral E and NS1 protein expression in BV2 cells 24 h post-infection. Immunostaining demonstrated significant viral dsRNA expression in DENV-infected BV2 cells (*p* < 0.05, Fig. 1E). A plaque assay, which was performed for determining viral replication and release, showed a significant (*p* < 0.05) successful infection of the BV2 cells by DENV (Fig. 1F). Finally, we demonstrated that the four DENV serotypes were capable of infecting BV2 cells based on the detection of viral E protein expression 24 h post-infection (Fig. 1G). These results indicate that DENV can infect microglia *in vitro*.

### DENV infection causes changes in cell morphology but not cell growth inhibition and cytotoxicity in microglia

**Figure 2:**
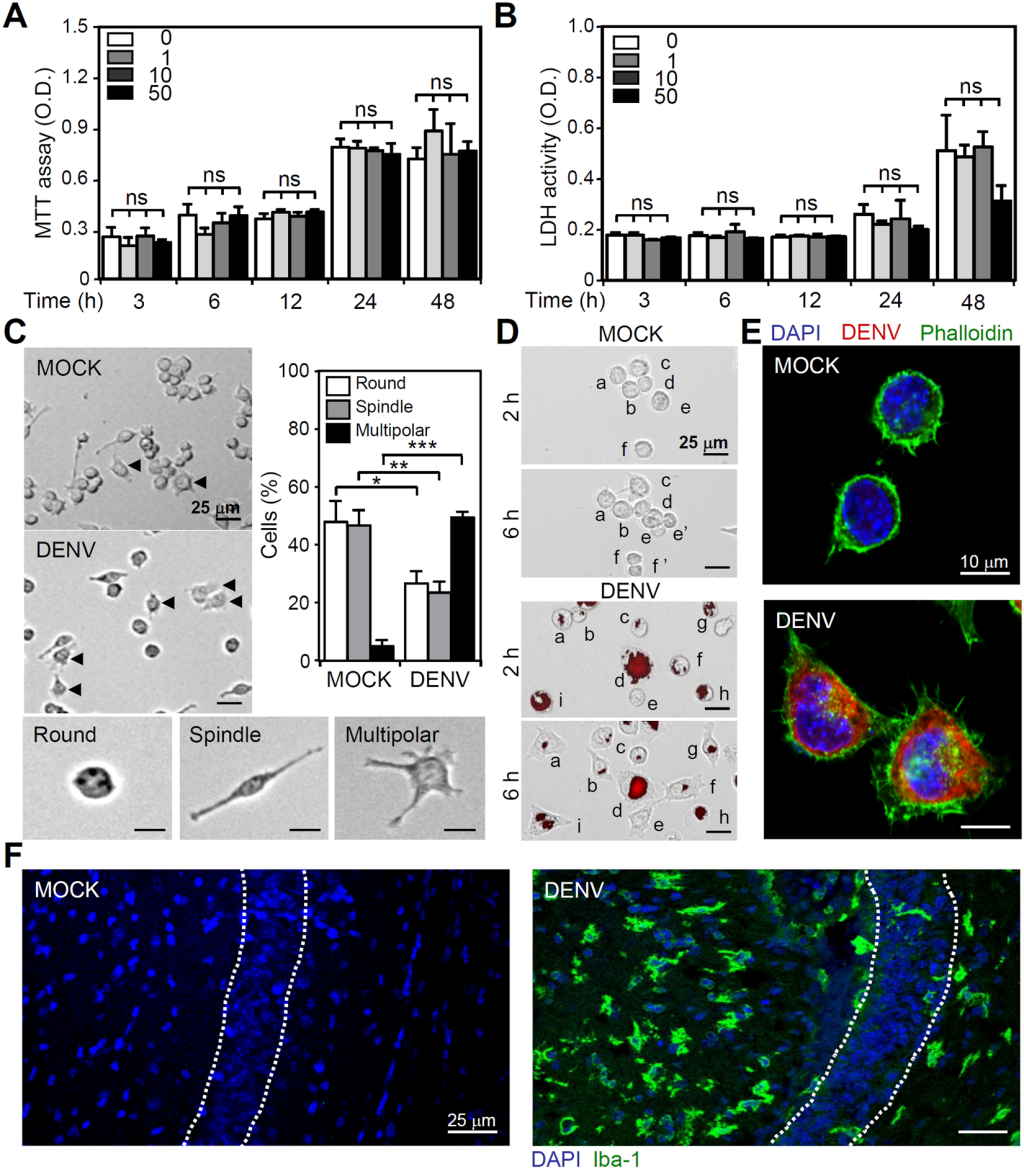
DENV infection causes minor effects on cell growth but considerably changes microglial cell morphology. (**A**) MTT and (**B**) LDH assays showed the cell viability and cytotoxicity, respectively, of BV2 cells inoculated with different MOIs of DENV 2 for the indicated different times. The quantitative data (optical density, O.D.) are shown as the means ± SD from three independent experiments. ns, not significant. The cells were inoculated with DENV 2 (MOI = 50) (**C**) without or (**D**) with Alexa-594 labeling (*red*). At 6 h post-infection, time-lapse microscopy showed changes in cell morphology characterized as round, spindle, and multipolar. The percentages of cells with morphological changes were determined and quantified as the means ± SD of three independent experiments. *p < 0.05, **p < 0.01, and ***p < 0.001. (**E**) Confocal microscopy showed actin polymerization (*green*) after DENV infection as detected by viral E protein expression (*red*). (**F**) Representative confocal fluorescent immunostaining of Iba-1 (*green*) in DENV-infected brains. Dotted lines indicate the hippocampal region. For all images, representative data were selectively obtained from three individual experiments. DAPI staining indicated the nuclei (*blue*).

Changes in cell growth, cytotoxicity, and morphology next evaluated the effects of DENV on microglial cells. The MTT (Fig. 2A) and LDH (Fig. 2B) assays showed that DENV did not cause cell growth inhibition and cytotoxicity 48 h post-infection, even when the cells were incubated with a high MOI. However, microscopic observation revealed a significant change in the microglial cells after DENV infection (Fig. 2C). The time-lapse microscopic observation showed that DENV infection resulted in increased formation of multipolar phenotypes compared with the MOCK cells. Fluorescent DENV-labeled microglial cells showed morphological changes towards the multipolar phenotype (Fig. 2D). Among these cells, motile features such as extended lamellipodia in a multipolar style were also detected (Fig. 2E). To verify the results of *in vitro* study, we have created an animal model of DENV infection in 7-day ICR suckling mice intraperitoneally and intracerebrally infected with DENV simultaneously^20^. In checking of immunofluorescent image of Iba-1 staining showed that DENV infection in the brains caused a significant morphological change on microglial cells toward active status characterized by multipolar phenotype *in vivo* among the infected hippocampal regions (Fig. 2F). These results indicate that changes occurred in microglial cell morphology toward migrating multipolar phenotype following DENV infection.

### DENV infection increases microglial migration

**Figure 3:**
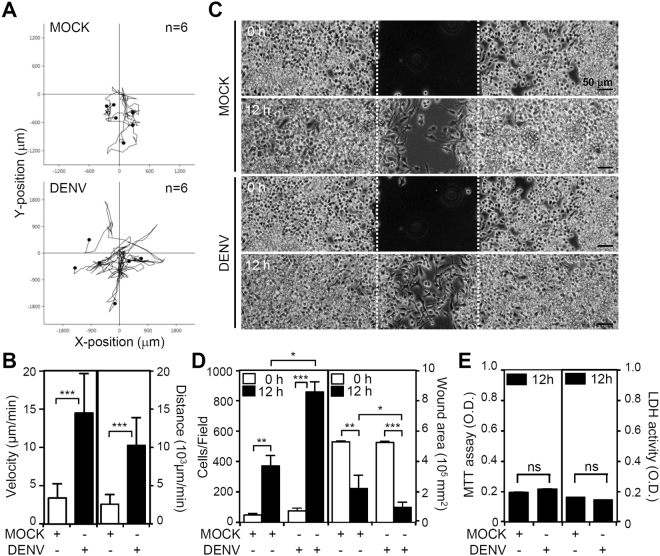
DENV infection increases microglial migration. (**A**) Time-lapse microscopy showed the kinetic tracking of cell migration in DENV 2-innoculated BV2 cells (n = 6) for 12 h. (**B**) Cell tracking analysis showed the velocity and distance of the migrated cells. (**C**) The wound-healing assay and (**D**) the measurement of the migrating cell number and wound area displayed cell migration. (**E**) The MTT and LDH assays showed cell viability and cytotoxicity, respectively, of cells inoculated with DENV. All quantitative data are shown as the means ± SD from three independent experiments. *p < 0.05, **p < 0.01, and ***p < 0.001. ns, not significant.

To assess whether the morphological changes towards the multipolar phenotype were necessary for cell migration, we performed a time-lapse microscopic observation. The microscopy results showed that DENV infection significantly (*p* < 0.05) increased microglial migration (Fig. 3A) not only in velocity but also in total distance (Fig. 3B). A wound healing assay analyzed whether DENV infection induced cell migration. The wound size and the number of migrating cells in the MOCK group were marginally different from these attributes in the DENV-infected cells 12 h post-infection (Fig. 3C). Following quantification, the number of migrating cells (Fig. 3D, left) and the re-coverage area of the wound (Fig. 3D, right) showed that DENV infection caused a significant increase in cell migration without affecting cell growth and cytotoxicity (Fig. 3E). Under the lower MOI of DENV infection, we also found an increase in microglial cell migration (Fig. S1). These results indicated that DENV infection had a microglial migration-promoting effect.

### Endocytosis-based viral entry contributes to the DENV-induced increase in microglial migration

**Figure 4:**
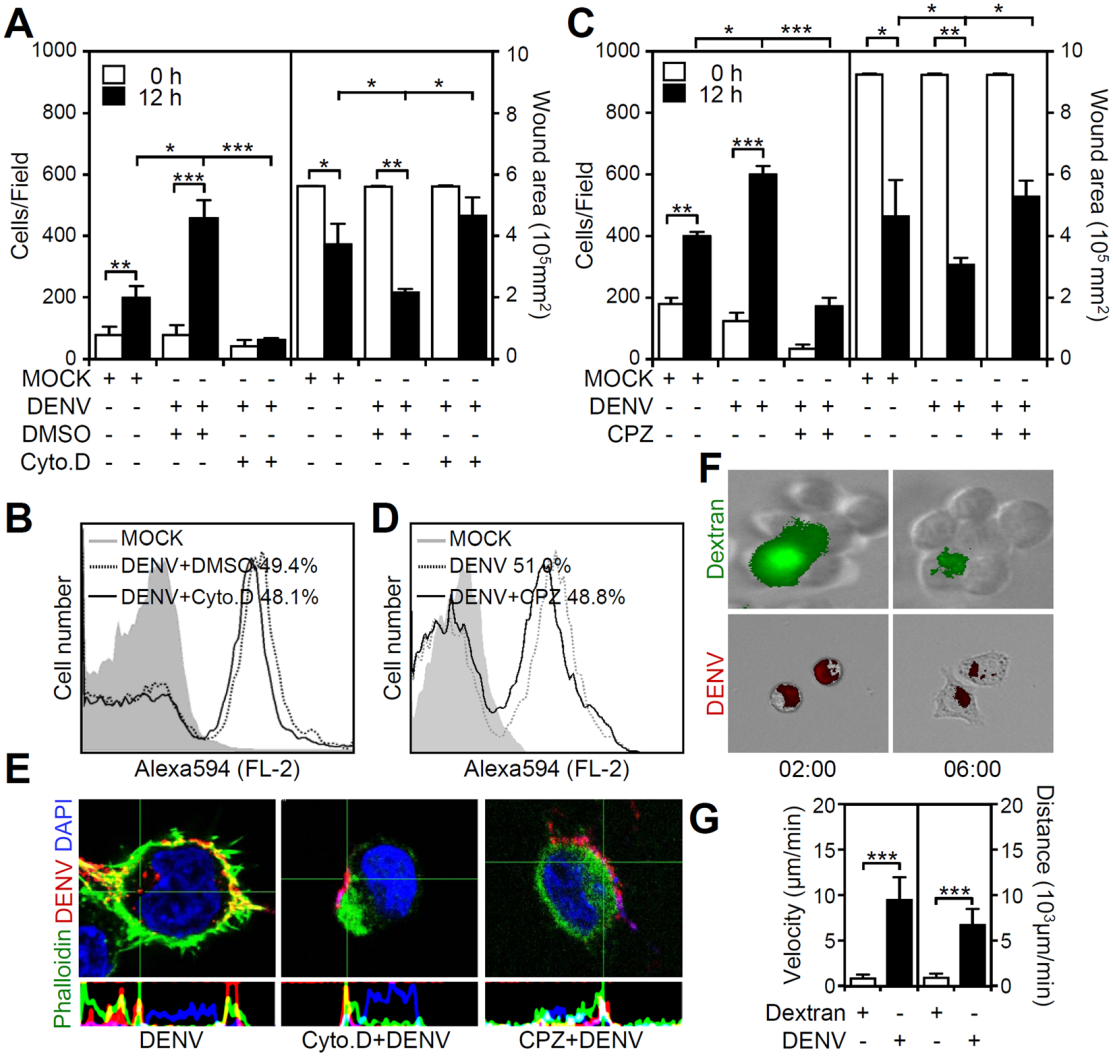
Inhibition of actin polymerization and clathrin-mediated endocytosis reduce the DENV-induced increase in microglial migration. BV2 cells were inoculated with (**A** and **C**) DENV 2 (MOI = 50) or (**B** and **D**) Alexa-594-labeled DENV 2 (MOI = 50) for 12 h in the presence of the actin polymerization inhibitor cytochalasin D (Cyto. D, 5 µM) and the clathrin inhibitor chlorpromazine (CPZ, 10 µg/ml). The numbers of migrated cells and wound area measurements showed cell migration. Flow cytometry were used to measure the percentage of microglial cells carrying fluorescent DENV. (**E**) Furthermore, confocal microscopy showed the binding/entry of Alexa-594-labeled DENV 2 (MOI = 50, *red*) in BV2 cells with or without Cyto. D and CPZ. Phalloidin (*green*) staining indicated actin. DAPI staining indicated the nuclei (*blue*). (**F**) BV2 cells were treated with dextran (*green*) or Alexa-594-labeled DENV 2 (*red*, MOI = 50) for 6 h. The time-lapsed images showed cell morphology changes. (**G**) Migrating cell tracking showed the velocity and distance of the migrated cells. For all images, representative data were selectively obtained from three individual experiments. The quantitative data are depicted as the means ± SD. *p < 0.05, **p < 0.01, and ***p < 0.001. ns, not significant.

To investigate the mechanisms underlying the DENV-induced microglial migration, we examined the involvement of phagocytosis (actin-mediated) and endocytosis (clathrin-mediated) using pharmacological approaches. First, we treated BV2 cells with an actin polymerization inhibitor to evaluate actin-dependent migration. The results demonstrated significant inhibition of DENV-induced cell migration by pre-treatment with cytochalasin D, which is an inhibitor of actin polymerization (*p* < 0.05, Fig. 4A). Actin mediates phagocytosis in macrophages and microglia. Flow cytometric analysis of the fluorescence-labeled DENV showed that inhibiting actin did not decrease viral binding onto and/or entry into microglial cells (Fig. 4B). Several studies have demonstrated that DENV enters cells via clathrin-mediated endocytosis^17^; however, studies have also shown that DENV entry is cell type dependent. Next, we examined the involvement of endocytosis (clathrin-mediated) using the pharmacological inhibitor chlorpromazine as previously described^21^. The wound healing study results (Fig. 4C) showed significant inhibition of the DENV-induced increase in cell migration following chlorpromazine pre-treatment (*p* < 0.05). However, flow cytometric analysis showed that inhibiting clathrin did not affect the percentages of microglia stained with fluorescence-labeled DENV (Fig. 4D). By using confocal image analysis, we found both inhibiting actin polymerization and clathrin-mediated endocytosis caused the blockade of DENV entry (Fig. 4E). To investigate the role of endocytosis in the regulation of microglial migration, we assessed dextran-based clathrin-mediated endocytosis^22^. However, dextran did not cause microglial cell morphological changes (Fig. 4F) or increase cell migration (Fig. 4G). These results show that DENV infection increases microglial migration through a mechanism involving clathrin-mediated endocytosis followed by the stimulation of viral and host factors.

### TLR3 signaling contributes to the DENV-induced increase in microglial migration

**Figure 5:**
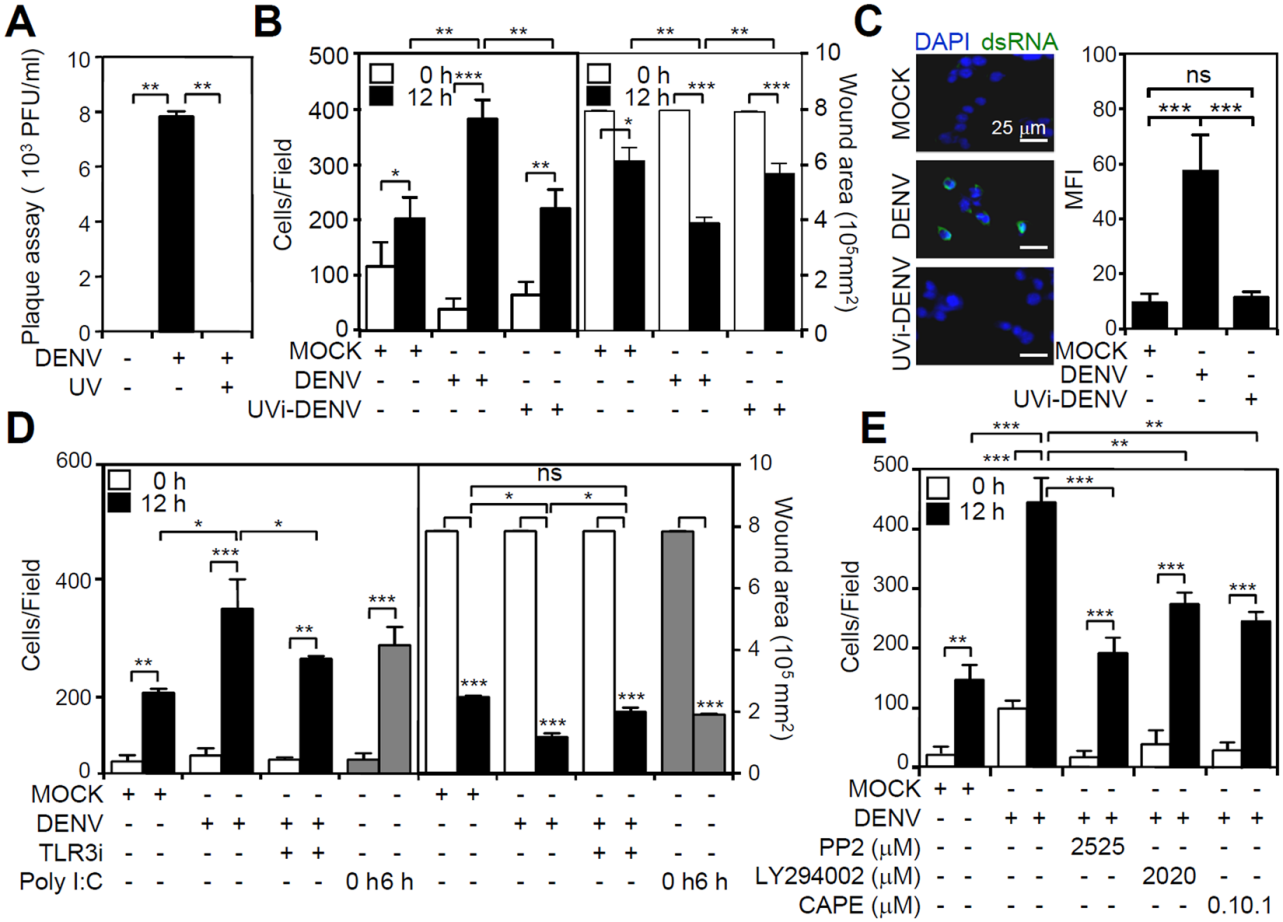
DENV infection increases cell migration through the TLR3/Src-regulated pathway. BV2 cells were inoculated with DENV 2 (MOI = 50) for 12 h in the absence or presence of UV irradiation (UVi). (**A**) The plaque assay confirmed virus release. (**B**) Wound-healing assay-based analysis of cell migration performed using the Image J software based on the number of migrating cells and wound area changes. (**C**) Representative fluorescent immunostaining images and MFI analysis displayed dsRNA replication. Migration in cells incubated with or without (**D**) TLR3 inhibitor (TLR3i, 5 µM) and (**E**) Src inhibitor (PP2, 25 µM), PI3K inhibitor (LY294002, 20 µM), or NF-kB inhibitor (CAPE, 0.1 μM) treatment was measured by the wound-healing assay 12 h post-infection. Poly(I:C) was used as a positive control for TLR3 activation. The quantitative data are depicted as the means ± SD. *p < 0.05, **p < 0.01, and ***p < 0.001. ns, not significant.

Because endocytosis did not affect microglial migration, we examined the effects of viral factors on migration. Following endocytosis, viral RNA is released into the cytoplasm followed by dsRNA replication^17^. Ultraviolet-inactivated (UVi) DENV did not cause viral replication and release in microglia (Fig. 5A) because UV irradiation of the virus broke down the viral RNA. In both cases, cell migration did not increase as shown by the wound healing assay (Fig. 5B). These results indicate that early viral replication is involved in triggering cell migration in microglia. Immunostaining confirmed that dsRNA replication was defective in microglial cells treated with UVi-DENV (Fig. 5C). Generally, dsRNA causes TLR3 activation to trigger innate immunity during DENV infection^23^. Pharmacologically inhibiting TLR3 significantly decreased DENV-induced activation of IFN regulatory factor 3, a downstream of TLR3 signaling (Fig. S2), as well as microglial migration (*p* < 0.05, Fig. 5D). Src, PI3K, and NF-κB are recruited to activate TLR3 and regulate cell migration^24, 25^. Therefore, we applied selective inhibitors of these proteins. All of inhibitions significantly reduced the DENV-induced activation of Src (Fig. S3), Akt (Fig. S4), and NF-κB (Fig. S5) as well as increase in microglial migration (Fig. 5E). These results demonstrate DENV infection causes an increase in microglial migration through a dsRNA-TLR3 signaling pathway.

## Discussion

The WHO^1^ recommends that new dengue cases be categorized as dengue without warning signs, dengue with warning signs, and severe dengue, which includes CNS involvement. In addition to DENV’s neuroinvasive capacity, prolonged shock, hyponatremia, hepatic failure, or intracranial bleeding may also cause neurological manifestations^3^. Clinical symptoms from patients with dengue encephalitis include diminished consciousness, headache, dizziness, disorientation, seizures, and behavioral symptoms. At present, the pathogenesis of dengue neurological complications and the underlying virus-host interactions remain undefined. The main target cell type for DENV infection in the brain is unknown, although DENV has been shown to infect microglial cells, which are the macrophage-like resident immune cells in the brain^5, 8^. However, no reports have shown the effects of DENV on microglial cells. In a recent study, we identified a potential role for microglia in facilitating the antiviral response by acting as antigen-presenting cells in the brain^20^. In this work, we demonstrate that microglial cells can be targets for DENV infection *in vitro*. DENV infection causes an increase in microglial migration without effecting cell viability and cytotoxicity.

Some receptors for DENV entry into cells have been demonstrated, such as heparin sulfate, CD14/LPS, DC-SIGN, GRP78, laminin receptor, heat shock proteins, beta3 integrin, mannose receptor, and C-type lectin domain family 5, member A (CLEC5A, MDL-1)^17^. Most DENV receptors are also expressed in microglia under differentiated stimulation conditions^26, 27^. Although our findings demonstrated that DENV infected microglia, the cell receptor(s) required for viral binding and entry during DENV infection is unknown. Similar to results obtained with other cell types, including C6/36, BHK21, and other mammalian cells^15, 17, 18^, we demonstrated that DENV entered microglial cells via a clathrin-regulated mechanism. Furthermore, we showed that clathrin-dependent viral entry was necessary for DENV-facilitated microglial migration. Because dextran treatment did not affect cell migration, cell receptors and/or viral factors may also be involved in stimulating microglial migration. Next, we demonstrated an essential role for dsRNA in the promotion of cell migration because UV-inactivated DENV did not have this effect. Therefore, the importance of the dsRNA- and TLR3-regulated signaling pathways, which were originally identified in DENV infection^23^, were confirmed in this study. dsRNA-activated TLR3 can trigger both gene transcription-dependent and independent pathways via recruitment of activated Src Tyr216 and PI3K^28^. Src and PI3K activation cause early activation of downstream kinases such as FAK, which is an essential mediator of various cellular activities (e.g., migration, adhesion and proliferation). NF-κB activation may facilitate cell migration through transcriptional regulation in chemokines, adhesion molecules, matrix metalloproteinases, and migration-promoting factors. In this study, pharmacologically blocking either TLR3 or TLR3-related signaling pathways reduced the DENV-induced increase in microglial migration. DENV also triggers TLR3-regulated cell migration in microglia, which is similar to TLR3 stimulation and activation in other contexts, such as influenza virus infection and dsRNA stimulation^24, 29^.

Microglia are the resident macrophages of the brain and spinal cord and maintain immune homeostasis in the brain by triggering scavenging, phagocytosis, cytotoxicity, antigen presentation, synaptic stripping, promotion of repair, and extracellular signaling^30^. During viral neuroinflammation, activated microglia change their morphology and alter their gene expression, leading to the production of numerous potentially neurotoxic mediators such as cytokines (IL-1α, IL-1β and TNF-α), chemokines (MCP-1, MIP-1α, and MIP-1β), proteases (cathepsins and MMPs), and amyloid precursor proteins^11^. Increased microglial migration caused by infections with viruses such as HIV may facilitate CNS viral infection and inflammation^31, 32^. A recent study demonstrated the induction of inflammatory cytokine/chemokine expression in DENV-infected microglia *in vitro* ^16^. According to these findings, activated microglia may be pathogenic in dengue encephalitis. However, pharmacologically depleting microglia exacerbates viral replication and DENV-induced acute viral encephalitis, indicating an immune defense role for DENV-infected microglia^20^. Our results also showed APC-like microglial differentiation that contributed to antiviral immunity in the brain during DENV infection. DENV infection also increases dendritic cell (DC) migration^33^. Therefore, the increased cell migration in DENV-infected microglia may mimic DENV-infected DCs. Indeed, virus-infected cells may play diverse roles in immunity, infection, and pathogenesis^34^. Taken together, these findings suggest that DENV infection not only induces APC-like differentiation in microglia but also increases cell migration similar to DENV-infected DCs.

Although severe dengue complications include CNS involvement and neurological dysfunction^3^, several questions need to be addressed to clarify the mechanisms underlying the pathogenesis of DENV-associated encephalitis. First, DENV neurotropism requires further investigation. In an *in vitro* study, we and others^16^ showed the ability of DENV to infect microglia; however, its effects, including inflammatory activation and an increase in cell migration, on neuroinflammation and neurotoxicity should be further studied. Additionally, it is important to verify the infectious ability of DENV in neuronal cells, particularly its direct effects on neuronal dysfunction. Second, creating an animal model of DENV-induced neuropathy is an important step to study DENV neuropathogenesis. An immunocompetent mouse model is necessary to assess the immune activation involved in CNS inflammation. Based on the successful infection of neonatal ICR mice by DENV demonstrated in our current study^20^ and others^9^, DENV causes infection and activation in microglial cells (Iba-1 positive) *in vivo*. Finally, it is essential to create an appropriate model of the peripheral route of DENV infection to mimic natural infection^8^. However, identifying the pathogenic mechanism of BBB disruption after DENV infection is necessary to dissect DENV-associated encephalitis.

**Figure 6:**
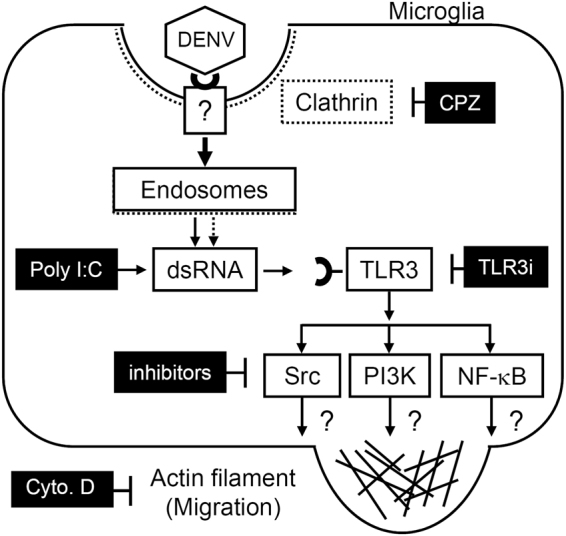
A hypothetical model of the DENV-induced increase in cell migration in microglia. Following infection through unknown receptor(s), DENV enters into microglial cells through clathrin-mediated endocytosis. dsRNA replication can activate the TLR3 signaling pathways to increase microglial migration.

In conclusion, our current works summarized in Fig. 6 not only confirmed the infectious ability of DENV in microglia but also showed an increased effect on the microglial migration induced by DENV. DENV bound to unknown receptors on the plasma membrane and triggered clathrin-mediated endocytosis. This process increased microglial migration via the TLR3-related signaling pathways. Migration-promoting factors induced by the infected cells, the binding or entry of virus particles, or the expression of virus proteins within the infected cells are probably involved in DENV-induced cell migration. Furthermore, migrating cells carrying virus may have potential effects on viral spreading, immune escape, and the antiviral response^34^. For other viruses of the *flaviviridae* family, more evidence is needed to clarify the pro-migratory effects of viral infection on microglial cells related to neurotoxicity. According to the findings of *in vitro* microglial cell migration caused by DENV-activated TLR3-regulated signaling pathways, it is speculated a blockade of microglial cell migration *in vivo* by targeting these pathways. However, several issues have to be resolved including a technique could be used to monitor cell migration in DENV-infected microglia *in vivo* and the strategies by targeting TLR3 signaling specially in DENV-infected microglia. After that, the effects of migrating microglia on virus infection, immune defense, CNS inflammation, and neurotoxicity remain unclear and need further investigation.

## Methods

### Antibodies and reagents

The reagents and antibodies (Abs) used were as follows: 4,6-diamidino-2-phenylindole (DAPI), dimethyl sulfoxide (DMSO), the PI3K inhibitor LY294002, the Src inhibitor PP2, the NF-κB inhibitor CAPE, and dextran (Sigma-Aldrich Co., St. Louis, MO, USA); cytochalasin D (Cyto. D) and chlorpromazine (CPZ) (Calbiochem, San Diego, CA, USA); the TLR3 inhibitor and Poly(I:C) (Tocris Bioscience, St. Louis, MO, USA); a mouse monoclonal Ab to β-actin (Chemicon International, Inc., Temecula, CA, USA); Alexa Fluor 488-conjugated goat anti-rabbit, Alexa Fluor 594-conjugated goat anti-mouse and Abs against the DENV NS1 and E proteins (GeneTex, Irvine, CA, USA); and Abs against dsRNA (Engscicons, Hungary). All drug treatments were assessed for cytotoxic effects using cytotoxicity assays prior to the experiments. Non-cytotoxic dosages were used in this study.

### Cell culture and virus culture

BV2 immortalized murine microglial cells were obtained from Dr. C. C. Huang (Department of Pediatrics, National Cheng Kung University, Tainan, Taiwan) and maintained using previously described conditions^35^. The cells were grown in Roswell Park Memorial Institute (RPMI) 1640 supplemented with 10% heat-inactivated fetal bovine serum (FBS) (Sigma-Aldrich), 50 U/ml of penicillin and 50 μg/ml of streptomycin in a humidified atmosphere with 5% CO_2_ and 95% air. Baby hamster kidney (BHK)−21 cells (ATCC® CCL-10™) and *Aedes albopictus* clone C6/36 cells (ATCC® CRL-1660™) were cultured in Dulbecco’s Modified Eagle Medium (DMEM, Invitrogen Life Technologies) containing FBS. The four DENV serotypes (DENV1 8700828, DENV2 PL046, DENV3 8700829A, and DENV4 59201818) were maintained in C6/36 cells. C6/36 cell monolayers were seeded in a 75-cm^2^ tissue culture flask with DENV inoculation at an MOI of 0.01 and incubated at 28 °C in 5% CO_2_ for 5 days. The virus supernatant was concentrated and filtered with Amicon Ultra centrifugal filters (Millipore, Billerica, MA, USA) and then stored at −80 °C prior to use. The virus titer was determined by plaque assay using the BHK-21 cell line. In general, a concentration of 10^7^ pfu/ml could be achieved from the standard preparation. To prepare the UV-inactivated virus, DENV was exposed to a 15 W UV lamp at a distance of 10 cm for 1.5 h. The viral supernatants were checked using plaque assays.

### DENV infection and plaque assay

BV2 cells were seeded at a density of 10^5^ cells per well in 6-well plates overnight, followed by the addition of DMEM with DENV (MOI of 50). The cells were incubated for 90 min at 37 °C. Then, the cells were washed once with medium and incubated at 37 °C with 5% CO_2_. The viral supernatants were checked using plaque assays. For the plaque assay, BHK-21 cells were plated in 12-well plates (2 × 10^5^ cells/well) and cultured in DMEM under CO_2_-enriched conditions. After adsorption with a serially diluted virus solution for 1 h, the solution was replaced with fresh DMEM containing 2% FBS and 0.5% methylcellulose (Sigma-Aldrich). Five days post-infection, the medium was removed and the cells were fixed and stained with a crystal violet solution containing 1% crystal violet, 0.64% NaCl, and 2% formalin.

### Fluorescent DENV

Fluorescent DENV was prepared by labeling with Alexa Fluor 594 succinimidyl ester (AF594SE, Molecular Probes, Invitrogen, Carlsbad, CA, USA) according to the method described in a previous study^36^. The labeled viruses were purified using Amicon Ultra-15 PLTK Ultracel-PL Membrane (30 kDa) centrifugal filter units (Millipore) to remove excess dye. After labeling, the DENVs were tested for fluorescence and stored in 50 μl aliquots at −80 °C prior to use.

### Immunostaining

To detect DENV2 E protein and dsRNA expression, the cells were fixed, stained, and analyzed as described elsewhere^35^. The cells were stained with primary Abs and then with the Alexa 488-conjugated goat anti-rabbit IgG and Alexa 594-conjugated goat anti-mouse IgG secondary Abs. DAPI (5 µg/ml) was used for nuclear staining. The cells were visualized under a fluorescent microscope (BX51; Olympus, Tokyo, Japan) or a laser-scanning confocal microscope (TCS SP5; Leica Mikrosysteme Vertrieb GmbH, Wetzlar, Germany). The three-dimensional images from a series of confocal images together with the z-axis of the cells and the analysis of the z-stacks were reconstructed using the Leica Confocal Software. For the flow cytometric analysis, the cells were stained with an anti-E Ab and then incubated with a mixture of Alexa Fluor 488-conjugated secondary Abs. The cells were analyzed using flow cytometry (FACSCalibur; BD Biosciences) with excitation set at 488 nm and emission detected with the FL-1 channel (515–545 nm). The samples were analyzed using the CellQuest Pro 4.0.2 software (BD Biosciences). The entry of labeled DENV into the cells was analyzed with a fluorescent microscope (BX51) and quantified using a FACS Canto II Flow Cytometer (BD Biosciences). For actin staining, the cells were seeded onto a coverslip and fixed, blocked, and stained with Alexa Fluor 594 phalloidin (Thermo Fisher Scientific, Pittsburgh, PA, USA).

### Western blotting

Harvested cells were lysed in a buffer containing 1% Triton X-100, 50 mM Tris (pH 7.5), 10 mM EDTA, 0.02% NaN_3_, and a protease inhibitor cocktail (Roche Boehringer Mannheim Diagnostics, Mannheim, Germany). After a freeze–thaw cycle, the cell lysates were centrifuged at 10,000 × g at 4 °C for 20 min. The lysates were boiled in a sample buffer for 5 min. Then, the proteins were subjected to SDS-PAGE and transferred to PVDF membranes (Millipore, Billerica, MA, USA) using a semi-dry electroblotting system. After blocking with 5% skim milk in PBS, the membranes were incubated overnight with 1:1,000 dilutions of primary Abs at 4 °C. The membranes were washed with 0.05% PBS-Tween 20 and incubated with a 1:5,000 dilution of a HRP-conjugated secondary Ab at room temperature for 1 h. After washing, the membranes were soaked in ECL solution (Perkin Elmer Life and Analytical Sciences, Inc., Boston, MA, USA) for 1 min and exposed to an X-ray film (BioMax; Eastman Kodak, Rochester, NY, USA). The relative signal intensity was quantified using the ImageJ software (version 1.41o; W. Rasband, National Institutes of Health, Bethesda, MD, USA). Changes in the protein ratios compared with the normalized values of untreated cells (indicated protein/β-actin) were also determined. One set of representative data obtained from three independent experiments is shown.

### Cell viability and cytotoxicity

Cell viability and cytotoxicity were assessed using MTT Cell Proliferation Kit (Sigma-Aldrich) and Cytotoxicity Detection kit assays (Roche Diagnostics, Lewes, UK), respectively, according to the manufacturer’s instructions. A microplate reader (SpectraMax 340PC; Molecular Devices Corporation, Sunnyvale, CA, USA) was used and the data were analyzed using the Softmax Pro software (Molecular Devices Corporation).

### Infectious animal model

Seven-day-old ICR suckling mice, purchased from Charles River Laboratories (Wilmington, MA) and cared for according to the guidelines established by the Ministry of Science and Technology, Taiwan, were used according to the rules of the Animal Protection Act of Taiwan, and all protocols were approved by the Laboratory Animal Care and Use Committee of National Cheng Kung University (IACUC #104062). The animals were inoculated with DENV2 (PL0146) by intracerebral (2.5 × 10^5^ pfu) and intraperitoneal (7.5 × 10^5^ pfu) injections simultaneously according to our previous works^20^.

### Immunofluorescence analysis

Mouse brains were prepared in tissue blocks and sliced. Sections (7 μm) were stained with antibodies against activated microglial marker Iba-1 (Abcam, Cambridge, MA). DAPI was used for nuclear staining.

### Wound-healing assay

A scratch wound was created using a Culture-Insert (BD Labware Europe, Le Pont De Claix, France). The BV2 cell monolayer (1.2 × 10^5^ cells/well) was seeded into a 24-well cell culture plate and cultured overnight. Then, the insert was removed, washed with PBS, and placed into fresh medium prior to virus infection. The wound area of the BV2 monolayer was photographed 0 or 12 h post-infection. The number of cells that migrated into the cleared wound area were counted using ImageJ (http://imagej.nih.gov/ij/download.html) and the reduction of the wound area was measured. The relative cell migration distance was analyzed using the Gradientech Tracking Tool (http://gradientech.se/tracking-tool-pro/)*.* The results were automated to track the cell migration distance and velocity.

### Time-lapse microscopy

The time-lapse fluorescent imaging of cell motility was monitoring using the Lumascope 620 microscope (Etaluma, Carlsbad, CA, USA). We utilized the Lumascope to obtain a time-lapse sequence of digital images taken every 10 min for 12 h. Alexa-labeled DENV particles were excited with a 473–491 nm laser. The fluorescent images were obtained with a 10x magnification objective.

### Statistical analysis

Data obtained from three independent experiments were presented as the mean ± standard deviation (SD). The statistical analysis was performed using Prism version 5 (GraphPad Software, San Diego, CA, USA). Two sets of data were analyzed using an unpaired Student’s t-test. Three or more sets of data were analyzed by one-way ANOVA with Tukey’s multiple comparison post hoc test. Statistical significance was set at *p* < 0.05.

## Electronic supplementary material

Supplementary Information
