# Supplementary material for: Dengue virus infection increases microglial cell migration
Source: Sci Rep. 2017 Mar 7;7:91. doi: 10.1038/s41598-017-00182-z (PMC5427823; doi:10.1038/s41598-017-00182-z)

Dengue virus infection increases microglial cell migration

Ming-Kai Jhan, Tsung-Ting Tsai, Chia-Ling Chen, Cheng-Chieh Tsai, Yi-Lin Cheng, Yi-Chao Lee, Chiung-Yuan Ko, Yee-Shin Lin, Chih-Peng Chang, Liang-Tzung Lin, and Chiou-Feng Lin

Supplemental Figure Legends

Figure S1 DENV infection increases migration in the microglial cell line BV2. Microglial BV2 cells were inoculated with DENV serotype 2 PL046 for 12 h. (**A**) The wound-healing assay and (**B**) the measurement of the migrating cell number and wound area displayed cell migration. All quantitative data are shown as the means ± SD from three independent experiments. *$p < 0.05$, **$p < 0.01$, and ***$p < 0.001$.

Figure S2 Inhibition of TLR3 reduces the DENV-induced IRF3 phosphorylation and nuclear translocation. BV2 cells were inoculated with DENV 2 (MOI=50) for 6 h in the presence of the TLR3 inhibitor (TLR3i). Representative immunostaining showed IRF3 phosphorylation at serine 396 (pIRF3, *green*) and nuclear translocation. DAPI staining indicated the nuclei (*blue*). Poly(I:C) was used as a positive control for TLR3 activation.

Figure S3 Inhibition of PI3K decreases the DENV-induced Akt phosphorylation. BV2 cells were inoculated with DENV 2 (MOI=50) for 6 h in the presence of the PI3K inhibitor LY294002. Western blotting analysis showed Akt phosphorylation at serine 473. The relative ratio to β-actin is shown.

Figure S4 Src inhibitor PP2 decreases the DENV-induced Src phosphorylation. BV2

cells were inoculated with DENV 2 (MOI=50) for 6 h in the presence of the Src inhibitor PP2. Western blotting analysis showed Src phosphorylation at tyrosine 416. The relative ratio to β-actin is shown.

Figure S5 NF-κB inhibitor CAPE decreases the DENV-induced NF-κB activation. BV2 cells were inoculated with DENV 2 (MOI=50) for 6 h in the presence of the NF-κB inhibitor CAPE. Representative immunostaining showed the nuclear translocation of NF-κB (*green*). DAPI staining indicated the nuclei (*blue*).

Jhan *et al.* Figure S1

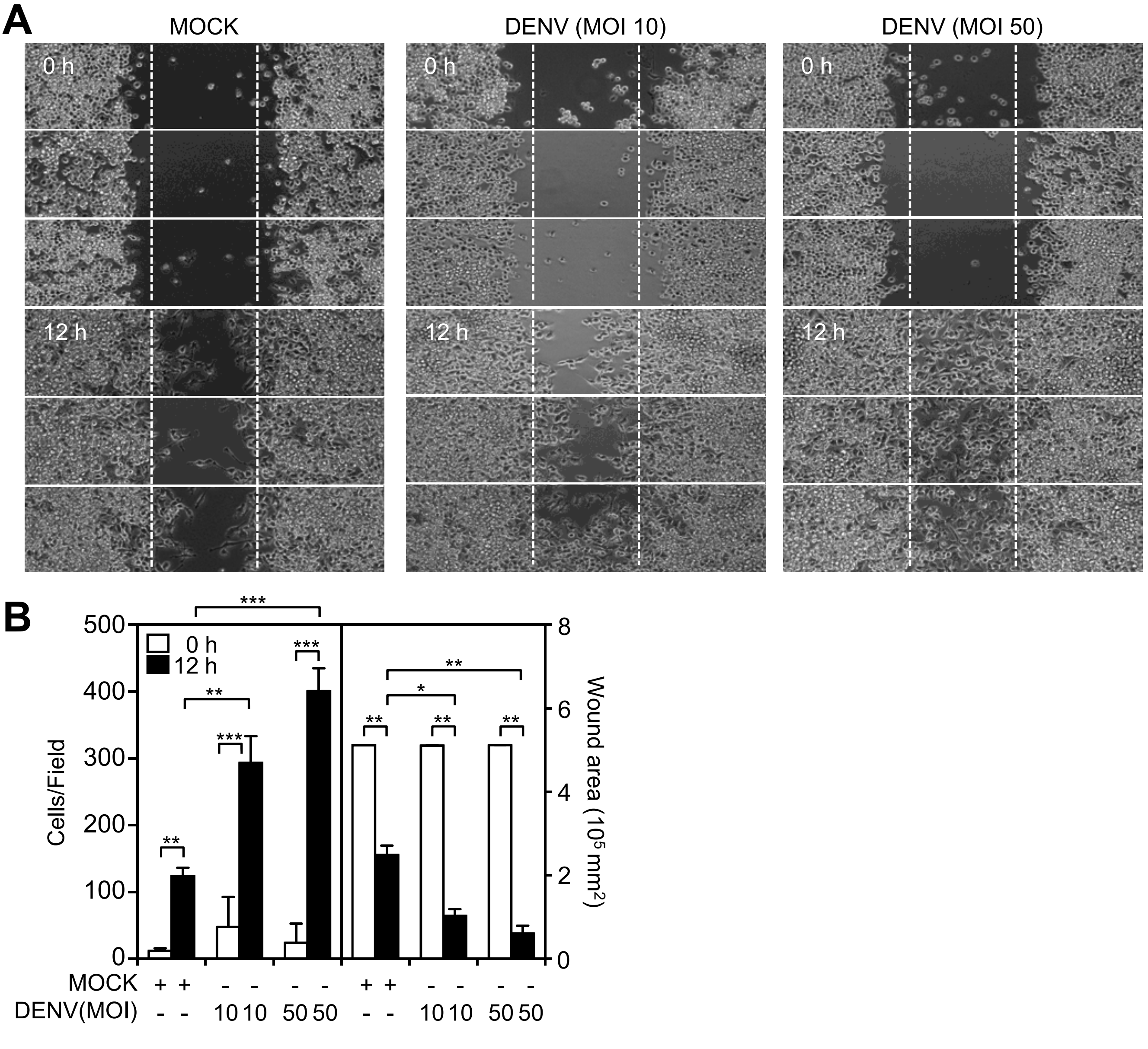

Jhan *et al.* Figure S2

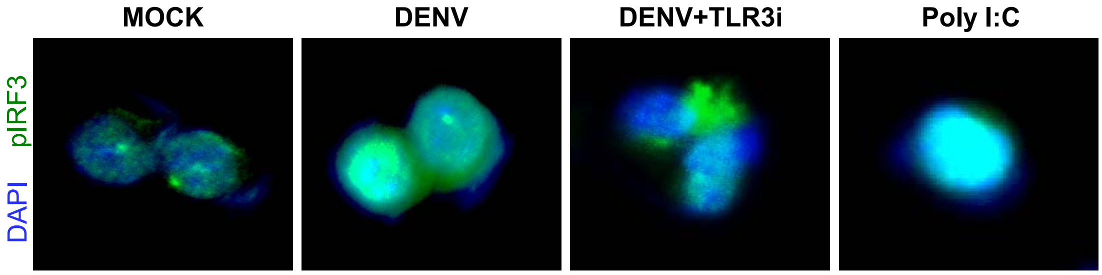

Jhan *et al.* Figure S3

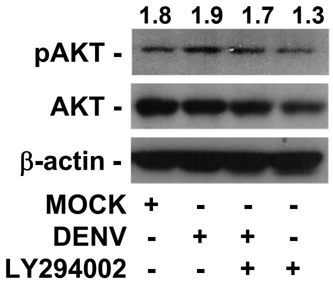

Jhan *et al.* Figure S4

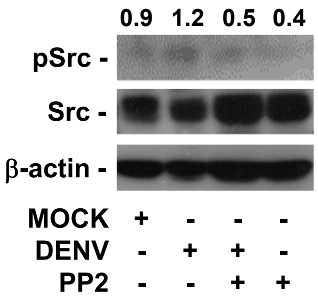

Jhan *et al.* Figure S5

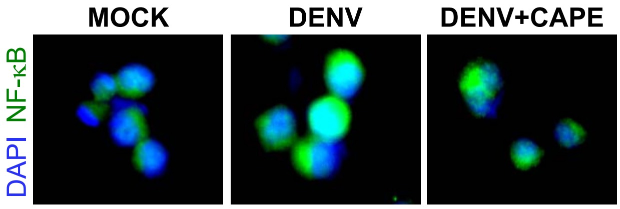

Supplement: Supplementary file 1 — Supplementary Information [file 41598_2017_182_MOESM1_ESM.pdf]
